# A predictive nomogram for intradiscal cement leakage in percutaneous kyphoplasty for osteoporotic vertebral compression fractures combined with intravertebral cleft

**DOI:** 10.3389/fsurg.2022.1005220

**Published:** 2022-10-05

**Authors:** Ning Fan, Tianyi Wang, Aobo Wang, Shuo Yuan, Peng Du, Fangda Si, Wenyi Zhu, Jian Li, Lei Zang

**Affiliations:** Department of Orthopedics, Beijing Chaoyang Hospital, Capital Medical University, Beijing, China

**Keywords:** cement leakage, intravertebral cleft, percutaneous kyphoplasty, osteoporotic vertebral compression fracture, nomogram

## Abstract

**Background:**

For patients with osteoporotic vertebral compression fractures (OVCFs) treated with percutaneous kyphoplasty (PKP), the occurrence and risk factors of intradiscal cement leakage should be characteristic of the presence of intravertebral cleft (IVC). This study aimed to identify risk factors for intradiscal leakage in individuals with OVCFs combined with IVC treated with PKP and build a powered and well-calibrated predictive nomogram.

**Methods:**

This study retrospectively reviewed consecutive patients who underwent PKP at our center between January 2016 and May 2021. Patients diagnosed with OVCFs combined with IVC were identified, and the incidence of different types of bone cement leakage was recorded. Risk factors for intradiscal leakage among the demographic, perioperative baseline, and radiologic data were identified, following which a nomogram was developed and verified.

**Results:**

A total of 109 eligible patients were included, and the intradiscal leakage rate was 32.1%. Compression rate (odds ratio [OR] 0.025; 95% confidence interval [CI] 0.002–0.264; *P* = 0.002) and cemented vertebral body fraction (OR 44.122; 95% CI 2.790–697.740; *P* = 0.007) were identified as independent risk factors. A predictive nomogram with good predictive power (C-statistic = 0.786) and fitness of data (Hosmer–Lemeshow goodness-of-fit test, *P* = 0.092) was established to build a quantitative relationship between the risk factors and intradiscal leakage.

**Conclusion:**

The incidence rate of intradiscal leakage in PKP for OVCFs combined with IVC was 32.1%. Compression rate and cemented vertebral body fraction were identified as independent risk factors. A powered and well-calibrated nomogram was established to accurately predict the probability of intradiscal leakage. Further prospective and multicenter studies are required to verify and calibrate our findings.

## Introduction

Osteoporotic vertebral compression fractures (OVCFs) are common among the elderly population and are characterized by pain, dysfunction, and loss of mobility and independence (1, 2). Conservative treatments, such as bracing, early mobilization, and osteoporotic treatment, have been proven effective for pain relief and functional improvement in most cases (2, 3). However, conservative treatment can still fail in certain individuals, causing persistent back pain and low quality of life (4). Recently, minimally invasive vertebral augmentation techniques, including percutaneous vertebroplasty (PVP) and percutaneous kyphoplasty (PKP), have been considered alternative options for the treatment of OVCFs (5–7). These procedures have the merits of partial vertebral height restoration and wedge deformity reversion (7). Although good clinical outcomes have been observed in most patients, these techniques are still associated with several complications.

Bone cement leakage (BCL) is one of the most common complications of vertebral augmentation techniques, the incidence of which varies from 7.9% to 79.9% (8–24). Traditionally, BCL is classified into three types: through the basivertebral vein (type B), through the segmental vein (type S), and through the cortical defect (type C) (25). However, a specific type, intradiscal leakage (type D), was distinguished from type C leakage by Tomé-Bermejo et al. (17). More attention should be paid to type D leakage, as it has been found associated with a higher incidence of adjacent vertebral fractures and subsequent pain (12, 26, 27).

Previous studies showed that the presence of intravertebral cleft (IVC) increases the incidence of intradiscal leakage after vertebral augmentation, which may attribute to the direct connection between the intervertebral disc space and intervertebral vacuum through endplate damage (13, 18, 20, 22). However, it still remains controversial and converse opinion was reported that the presence of IVC had no effect or even preventive effect to intradiscal leakage (12, 17, 28). A reasonable theory is that the cystic cavity in the vertebrae could promote a more homogeneous and controlled filling of the fractured vertebral body, decreasing the pressure of bone cement and risk of leakage (17).

The incidence of intradiscal leakage is low after PKP, and the reason is similar to the abovementioned theory that the inflatable balloon can create an iatrogenic cystic cavity-like space (9, 19, 29, 30). Nevertheless, there were still cases of intradiscal leakage, as high as 15.2%–22.6%, after PKP in some studies (11, 12, 24). In addition, interestingly, the preventive efficacy of intradiscal leakage seemed not to be strengthened by the presence of IVC in patients with OVCFs treated with PKP, and an even higher leakage rate was reported (11, 24). The exact reason for this remains unclear, and it is of great interest to determine whether there are specific triggers that balance these two theories.

Therefore, this study aimed to identify risk factors for intradiscal leakage and build a powered and well-calibrated predictive nomogram in individuals with OVCFs combined with IVC treated with PKP, to further explore clinical strategies to prevent intradiscal leakage in such patients.

## Methods

### Patient population

This study retrospectively reviewed consecutive patients who underwent PKP at our center between January 2016 and May 2021. The inclusion criteria were as follows: (1) age >55 years; (2) diagnosis of OVCFs from T7 to L5 based on evidence shown on preoperative radiography or CT and MRI (performed within 2 weeks before surgery); (3) severe back pain aligned with imaging tests; and (4) clear diagnosis of IVC. IVC was identified as a transverse, linear, or cystic region of gas-like hypointensity in the collapsed vertebral body shown on MRI T1-weighted sequences and hyperintensity on MRI T2 short-tau inversion recovery sequences (17, 28, 30). To control for confounding factors, we excluded patients who met the following exclusion criteria: (1) previous spinal surgery; (2) multilevel PKP; (3) preoperative tumor, infection, or deformity; and (4) incomplete data. This study was approved by the institutional review board.

### Surgical technique

PKP was performed in the prone position under local anesthesia, and all of the procedures were conducted by a unilateral transpedicular approach. A needle and inflatable balloon (Medtronic Sofamor Danek, Memphis, TN, United States) were inserted through the working channel into the fractured vertebral body under visualization with lateral and anteroposterior fluoroscopy. Then, a kyphoplasty balloon was used to inflate and create the cavity. Subsequently, the balloon was deflated, removed, and filled with viscous polymethylmethacrylate (Mendec Spine Cement; Tecres SPA, Verona, Italy) under fluoroscopic guidance. The procedure was stopped immediately once BCLs were detected. The surgical time and cement volume were recorded.

### Imaging evaluation and risk factors

**Figure 1:**
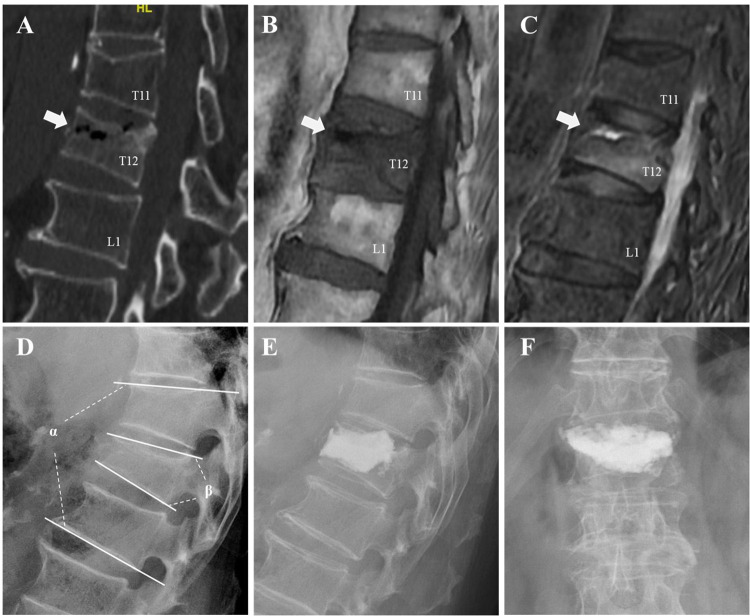
An illustrative case of an 81-year-old male patient underwent intradiscal leakage after PKP for OVCFs combined with IVC. A fracture in T12 was detected and the presence of an IVC (the white arrows) was confirmed by a gas-like density shown on preoperative CT (**A**), hypointensity shown on preoperative MRI T1-weighted sequences (**B**), and hypointensity shown on MRI T2 short-tau inversion recovery (**C**). Preoperative segmental kyphotic angle (*α*) and vertebral wedge angle (*β*) were measured on lateral radiograph (**D**). Intradiscal leakage was identified by postoperative radiographs (**E,F**).

BCL was assessed by postoperative radiography or CT, which were performed within three days after PKP. BCL was defined as the presence of extravertebral cement (Figure 1). Furthermore, BCL was classified into four types based on the location of the extravertebral cement according to Tomé-Bermejo et al. (17): (1) through the basivertebral vein (type B), (2) through the segmental vein (type S), (3) through the cortical defect and extraosseous non-intradiscal (type C), and (4) intradiscal leakage (type D).

Potential risk factors for intradiscal leakage in PKP for OVCFs combined with IVC were divided into two categories: First, demographic and perioperative baseline data included age, sex, weight, body mass index (BMI), overweight, obesity, time before surgery, bone mineral density (BMD), surgical level, and cement volume. Second, radiologic data included fracture type, fracture severity, presence of endplate cortical disruption, location of IVC, preoperative vertebral wedge angle, segmental kyphotic angle, minimum vertebral height, compression rate (CR), and cemented vertebral body fraction (CVBF).

Fractures were divided into three types based on a visual semiquantitative classification (31): wedge, biconcave, and crush type. According to the percentage of vertebral body collapse modified by Nieuwenhuijse et al. (18), fracture severity was graded into the following four levels: mild (20%–25%), moderate (25%–40%), severe (40%–67%), and very severe (>67%). The location of IVC was classified as follows: adjacent to the superior endplate, adjacent to the inferior endplate, and extending to both endplates (32).

All imaging parameters were evaluated on lateral radiographs. Vertebral wedge angle was measured as the angle between the superior and inferior endplates of the fractured vertebra, and segmental kyphotic angle was measured as the angle between the superior and inferior endplates of the two adjacent vertebrae. Minimum vertebral height was defined as the minimum height of the fractured vertebra. CR was calculated as fractured vertebral minimum vertebral height divided by the average vertebral heights of the two adjacent vertebrae (10). Furthermore, we measured CVBF to determine the individual efficacy of the cement injection volume. CVBF was calculated as the ratio of CV to fractured vertebral volume (33). Fractured vertebral volume was calculated by volume reconstruction of DICOM files of preoperative CT axial images using *Mimics* 21.0 (Materialize, Leuven, Belgium).

### Statistical analysis

Univariate and multivariate analyses were conducted using *SPSS* 24.0 (IBM, Armonk, NY, United States). Potential risk factors for intradiscal leakage were divided into demographic, perioperative baseline, and radiologic data. First, Student t-test or Mann–Whitney U test for continuous variables, and chi-square tests or Fisher exact tests for categorical variables were used for univariate analysis. Next, potential risk factors (*P* < 0.10 in univariate analysis) were included in the logistic regression model, and the stepwise forward method was performed for multivariate analysis. Statistical significance was set at a *P*-value <0.05. Finally, a nomogram was built as a predictive model based on logistic regression analysis using *R* 4.1.2 (R Foundation for Statistical Computing, Vienna, Austria). Receiver operating characteristic (ROC) curves were drawn, and C-statistics were calculated to determine the predictive power of the logistic regression model and nomogram. The calibration curve and Hosmer–Lemeshow goodness-of-fit tests were used to evaluate the fitness of the data.

## Results

A total of 109 eligible patients (40 men and 69 women) were included in this study. The mean age of the enrolled patients was 77.5 ± 7.9 years. The most common surgical levels were the thoracolumbar vertebrae (83.5%), followed by the lumbar vertebrae (11.0%) and thoracic vertebrae (5.5%). The median time from injury to surgery was 44 days. A total of 100 (91.7%) patients had endplate cortical disruption. Overall, 41 (37.6%) patients had BCLs, including 35 (32.1%) with type D, 7 (6.4%) with type S, 5 (4.6%) with type C, and 2 (1.8%) with type B (Table 1).

**Table 1:** Clinical baseline characters.

Clinical baseline characters (*N* = 109)	Mean ± SD or *N* (%)
Age, years	77.5 ± 7.9
Gender (female), *n*	69 (63.3%)
Surgical level, *n*	
Thoracic (upper than T10)	6 (5.5%)
Thoracolumbar (T10–L2)	91 (83.5%)
Lumbar (lower than L2)	12 (11.0%)
Endplate cortical disruption, *n*	100 (91.7%)
Time before surgery, days^a^	44 (38–60)
BLC, *n*^b^	41 (37.6%)
Type B	2 (1.8%)
Type S	7 (6.4%)
Type C	5 (4.6%)
Type D	35 (32.1%)

SD, standard deviation; BCL, bone cement leakage.

^a^ Results are given as the median (interquartile range).

^b^ Sum of different types is not equal to overall BLC because there are patients identified more than one type of leakage.

Among demographic and perioperative baseline data, CV (6.0 [5.5–7.5] ml vs. 4.5 [3.0–6.1] ml, *P* < 0.001) was found to be significantly higher in the intradiscal leakage group than in the control group. Age, sex, weight, BMI, overweight or obesity, time from injury to surgery, BMD, and surgical level did not significantly differ between the two groups (Table 2). In radiologic data, there were significant difference in fracture severity (*P* = 0.008), with more severe fracture and fewer mild fracture in the intradiscal leakage group. Also, we found lower minimum vertebral height (12.3 ± 3.7 vs. 15.6 ± 5.1, *P* = 0.001), lower CR (51.8 ± 18.2 vs. 66.8 ± 19.1, *P* < 0.001), and higher CVBF (35.0 ± 15.9 vs. 23.8 ± 15.3, *P* = 0.001) in the intradiscal leakage group (Table 3). Although all patients in the intradiscal leakage group had existing endplate cortical disruption, the incidence did not significantly differ from that in the control group (35/35 [100%] vs. 65/74 [87.5%], *P* = 0.075). 9 patients were found without endplate cortical disruption in the control group, including 5 combined with cortical disruption in anterior wall, 2 combined with cortical disruption in posterior wall, and 2 without any cortical disruption.

**Table 2:** Univariate analysis of demographic and perioperative baseline data for intradiscal leakage.

Variable	Intradiscal leakage group (*N* = 35)	Control group (*N* = 74)	*P* value
Age, year	78.0 ± 9.0	77.3 ± 7.3	0.635
Gender (female), *n*	22 (62.9%)	47 (63.5%)	0.947
Weight, kg	62.6 ± 12.8	62.5 ± 12.1	0.972
BMI, kg/m^2^	24.4 ± 4.7	23.4 ± 3.7	0.209
Overweight (BMI 25–30), *n*	15 (42.9%)	23 (31.1%)	0.228
Obesity (BMI ≥ 30), *n*	4 (11.4%)	2 (2.7%)	0.157
Time before surgery, days*	50 (39–80)	43 (38–60)	0.128
BMD, T-score	−3.2 ± 0.6	−3.0 ± 0.7	0.587
Surgical level, *n*			0.855
Thoracic (upper than T10)	2 (5.7%)	4 (5.4%)	
Thoracolumbar (T10–L2)	30 (85.7%)	61 (82.4%)	
Lumbar (lower than L2)	3 (8.6%)	9 (12.2%)	
CV, ml*	6.0 (5.5–7.5)	4.5 (3.0–6.1)	**<0.001**

BMI, body mass index; BMD, bone mineral density; CV, cement volume.

Bold values indicate statistical significance (*P* < 0.05).

* *P* values were calculated *via* the Mann–Whitney *U* test and results are given as the median (interquartile range).

**Table 3:** Univariate analysis of radiologic data for intradiscal leakage.

Variable	Intradiscal leakage group (*N* = 35)	Control group (*N* = 74)	*P* value
Fracture type, *n*			0.102
Wedge	13 (37.1%)	31 (41.9%)	
Biconcave	22 (62.9%)	38 (51.4%)	
Crush	0 (0)	5 (6.8%)	
Fracture severity, n*			**0** **.** **008**
Mild**	0 (0)	9 (12.2%)	
Moderate	9 (25.7%)	25 (33.8%)	
Severe**	18 (51.4%)	21 (28.4%)	** **
Very severe	5 (14.3%)	5 (6.8%)	
Endplate cortical disruption, n	35 (100%)	65 (87.8%)	0.075
Location of ICV, *n*			
Adjacent to superior endplate	17 (48.6%)	34 (45.9%)	0.798
Adjacent to inferior endplate	3 (8.6%)	11 (14.9%)	0.542
Extending to both endplates	12 (34.3%)	20 (27.0%)	0.437
VWA, °	13.3 ± 6.6	12.1 ± 7.4	0.397
SKA, °	17.6 ± 13.5	16.2 ± 15.4	0.657
VHmin, cm	12.3 ± 3.7	15.6 ± 5.1	**0**.**001**
CR, %	51.8 ± 18.2	66.8 ± 19.1	**<0**.**001**
CVBF, %	35.0 ± 15.9	23.8 ± 15.3	**0**.**001**

IVC, intravertebral cleft; VWA, vertebral wedge angle; SKA, segmental kyphotic angle; VHmin, minimum vertebral height; CR, compression rate; CVBF, cemented vertebral body fraction.

Bold values indicate statistical significance (*P* < 0.05).

* 17 patients were excluded because their vertebral body collapses were less than 20%.

** Indicates significant difference between pairwise comparison (Bonferonni adjusted *P* < 0.05).

To build a logistic regression model, we selected cement volume, fracture severity, endplate cortical disruption, minimum vertebral height, CR, and CVBF as potential risk factors (*P* < 0.1). However, we excluded cement volume, fracture severity, and minimum vertebral height as they showed significant collinearity with others and had negative effects on model prediction. Stepwise forward binary logistic analysis revealed that CR (odds ratio [OR] 0.025; 95% confidence interval [CI] 0.002–0.264; *P* = 0.002) and CVBF (OR 44.122; 95% CI 2.790–697.740; *P* = 0.007) were independent risk factors (Table 4).

**Table 4:** Multivariate logistic analysis for intradiscal leakage.

Variable	OR	95% confidence interval	*P* value
Endplate cortical disruption	–	–	0.099
CR	0.025	0.002–0.264	**0** **.** **002**
CVBF	44.122	2.790–697.740	**0**.**007**

OR, odds ratio; CR, compression rate; CVBF, cemented vertebral body fraction.

Bold values indicate statistical significance (*P* < 0.05).

**Figure 2:**
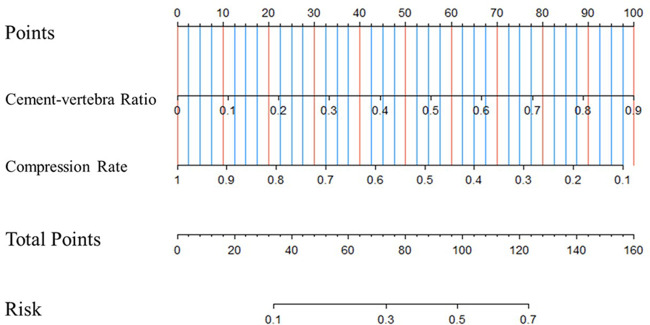
Predictive nomogram for intradiscal leakage in osteoporotic vertebral compression fractures combined with intravertebral cleft treated by percutaneous kyphoplasty.

**Figure 3:**
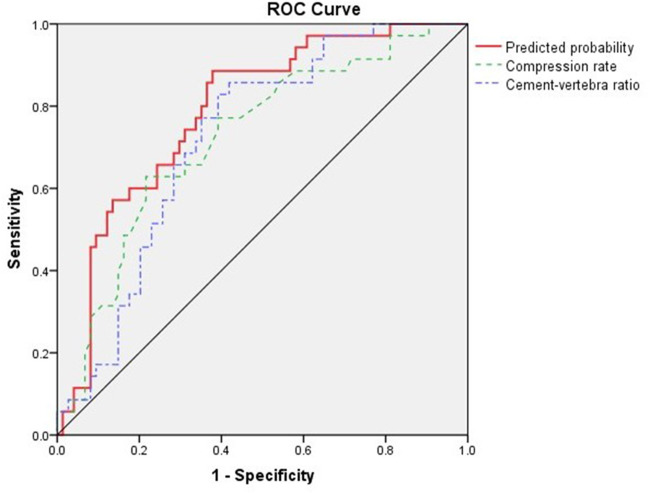
Receiver operating characteristic (ROC) curve of compression rate, cemented vertebral body fraction, and overall predicted probability, C-statistic = 0.786.

**Figure 4:**
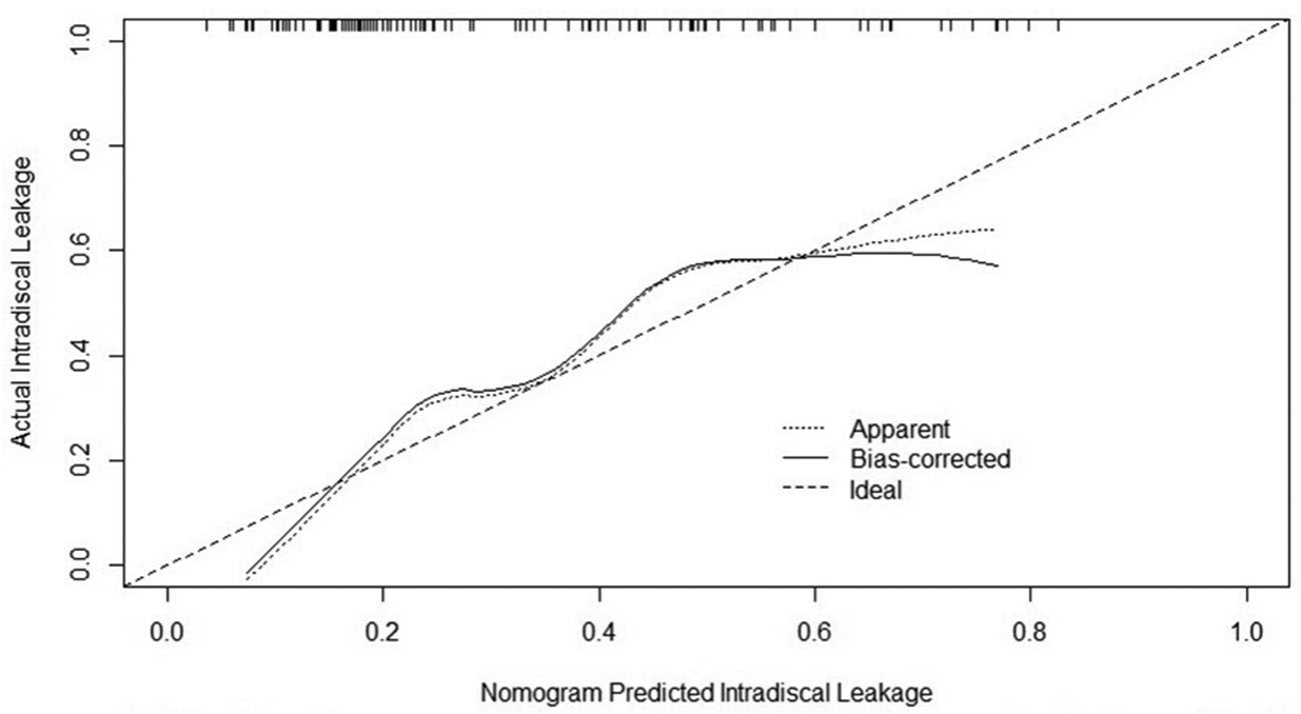
Calibration curve of the nomogram showed a well-calibrated predictive model, Hosmer–Lemeshow goodness-of-fit test *P* = 0.092.

Based on the results of the multivariate logistic analysis, a predictive nomogram was established (Figure 2). CR and CVBF, as independent risk factors, were scored, and a quantitative relationship with intradiscal leakage was built in patients with OVCFs combined with IVC treated with PKP. Then, the ROC curves of CR, CVBF, and overall predicted probability were drawn and showed good predictive power (C-statistic = 0.786) for the multivariate logistic model and nomogram (Figure 3). The calibration curve showed good fitness of the data and a well-calibrated predictive model (Hosmer–Lemeshow goodness-of-fit test *P* = 0.092; Figure 4).

## Discussion

For patients with OVCFs treated with PKP, the occurrence and risk factors of intradiscal cement leakage should be characteristic of the presence of IVC. However, to the best of our knowledge, the present study is the first to provide a unique insight into identifying risk factors for intradiscal leakage among such specific individuals. Our results revealed that the incidence of overall BCLs was 37.6%, whereas intradiscal leakage was the most common type, which developed in 32.1% of patients. CR and CVBF were identified as independent risk factors, and a powered and well-calibrated predictive nomogram was established to further explore the clinical strategies to prevent intradiscal leakage in such patients.

The presence of IVC may have conflicting effects on the different types of BCLs. Several studies have indicated that IVC decreases the risk of leakage through the veins (types B and S) (17, 28, 34, 35), and the effects may be multifactorial. First, IVC is caused by avascular osteonecrosis, and the area is surrounded by a fibrocartilaginous membrane, which makes the cement hard to extrude into the paravertebral veins (28). Moreover, pathological evidence showed that the occlusion of segmental arteries caused by fracture fragments and poor vascular supply beneath the superior endplate, where most IVCs occur, both contributed to a lower probability of venous leakage (36). In contrast, previous studies have recognized that the presence of IVC increases the risk of discal leakage, mainly because most clefts are connected directly between the intravertebral cavity and intradiscal area through the disrupted endplate, providing a low stress approach for cement distribution (13, 18, 20, 22, 31, 37, 38). In agreement with these findings, this study also found a lower incidence of 8.3% in venous leakage compared to as high as 32.1% in discal leakage in OVCFs combined with IVC.

Interestingly, in a recent study on BCLs after PVP (34), although a similar conclusion was drawn that IVC had opposite impacts on leakage through the vein and bone cortex, we noticed that the overall incidence of venous leakage was still higher than that of discal leakage (37.4% vs. 16.5%), contrary to our results. The authors believe that this is mainly because PKP exacerbates this contradiction. On the one hand, the inflated balloon may cause more occlusion or damage to the vascular system, leading to less cement leakage into the veins. On the other hand, the ballooning procedure may play a jack-like role when IVC is present, which results in pushing the normal bone apart to aggravate the cleft instead of compressing the bone (39). Together with the presence of a fibrocartilaginous membrane, the cement will not form an interdigitated but a crumby distribution and will leak into the disc through the increased cleft when filling the vacuum. However, these findings should be verified in further anatomical and pathophysiological studies.

In this study, the incidence of type D leakage was higher than 3.7%–18.0% reported by previous studies of PKP for treatment of OVCFs with IVC (11, 24, 29). We believe that there are two main reasons for this. First, cortical disruption has generally been identified as a risk factor for intradiscal leakage (10, 12, 13, 18, 37). Of the patients enrolled in our study, 91.7% had endplate cortical disruption and 97.0% were found to communicate with the IVCs. Second, approximately 45.0% of patients had severe or very severe fractures, yet the mean CV (5.5 ± 2.8 ml) was relatively large, which may account for the high incidence of intradiscal leakage. Wang et al. suggested that meticulous expansion of the balloon and filling with cement could prevent the risk of cement leakage to some extent (30). However, these procedures were difficult to perform in our experience, especially for patients with severe fractures, as we should balance well between the maximum restoration of vertebral height and prevention of BCLs for better prognosis, and the threshold was difficult to identify. Therefore, further identification of quantitative predictors of intradiscal leakage is of great benefit and requirement.

Previous studies have evaluated the relationship between fracture severity and intradiscal leakage after vertebral augmentation techniques (10, 12–14, 17, 18). Generally, the measurements of the severity of OVCFs can be divided into semiquantitative methods (17, 31) and quantitative parameters, such as fractured vertebral height and CR. In a retrospective study of 283 vertebrae in 239 patients with OVCFs, fracture severity was not recognized as a risk factor for intradiscal leakage (12). However, most studies have identified severe fracture as an independent risk factor (10, 13, 14, 17, 18). Similarly, the present study comprehensively evaluated semiquantitative and quantitative parameters of fracture severity and found that CR was the only independent risk factor. This can be explained by more severe vertebral fractures aligned with more endplate destruction, which may shorten the path between the IVC cavity and the destroyed endplate. In addition, severe vertebral fractures result in a less volume of vertebra, which limits the potential of filling the cement and increases the risk of cement leakage (10).

The role of the injected cement volume in intradiscal leakage remains conflicting and unclear (10, 12, 14, 26, 40). Chen et al. found that a greater amount of injected cement resulted in a higher tendency for cement leakage in the disc during PVP (26). Similarly, in a 10-year retrospective study of 485 patients, Zhu et al. identified that lower cement volume had a protective effect against intradiscal leakage in PVP (14). However, an association between cement volume and intradiscal leakage has not been found in other studies (10, 12, 40). The authors believe that the inconsistency may partly be attributed to the fact that these studies did not adjust cement volume to a specific vertebral volume in different individuals. For instance, an amount of 4.5 ml cement volume has different effects among different vertebral sizes, as a small and severely fractured vertebra was not likely to contain such a cement volume, thus leading to cement leakage. Therefore, the present study used CVBF, a vertebral volume-adjusted parameter, in the risk factor analysis and identified it as an independent risk factor for intradiscal leakage. The cavity area of the IVC and inflated balloon was limited by vertebral volume. When the cement fills a finite space, it tends to leak through the path from the IVC to the destroyed endplate, causing intradiscal leakage.

It is generally believed that endplate cortical disruption is a crucial risk factor for intradiscal leakage (10, 12, 13). However, although slightly more endplate cortical disruptions were found in intradiscal leakage group in this study, the difference did not reach statistical significance (35/35 [100%] vs. 65/74 [87.8%], *P* = 0.075). The main reason for this may because all patients enrolled in this study was with IVC, while IVC were found to be communicated with the endplate cortical disruption in 89.0% patients. This resulted in a high incidence of endplate cortical disruption, which may weaken its effect on contributing to the discrepancy in the two groups. Moreover, Tang et al. demonstrated that all intradiscal cement leaks were occurred through the cortical disruption at the endplates (35). This was confirmed by this study, and we also found all endplate cortical disruptions were communicated with IVC in intradiscal leakage group. Therefore, we inferred that endplate cortical disruption may be a requisite for intradiscal leakage in patients with IVC, rather than just a risk factor. However, we cannot draw an arbitrary conclusion, and further pathophysiological studies were required.

The present study attempted to build a novel nomogram to quantitatively predict the risk of discal leakage of PKP for the treatment of OVCFs with IVC. Our nomogram, containing CR and CVBF, showed a good predictive value (C-statistic = 0.786) and good fitness of data (Hosmer–Lemeshow goodness-of-fit test *P* = 0.092). Among the two independent risk factors, CR is an intrinsic risk factor that is non-modifiable since injury, whereas CVBF is a modifiable risk factor. This allows surgeons to calculate the most suitable threshold of injected cement volume according to CR to further reduce the risk of discal leakage in such individuals.

This study has some limitations. First, this was a retrospective and single-center study, which may have led to a selection bias. Second, the study had a relatively small sample size and the outcome of the intradiscal leakage group was limited to an even smaller sample size of 35 patients. However, the number of events per variable included in the logistic regression model should be greater than 10, which indicates that the multivariate model was sufficiently stable in this study. Moreover, several potential risk factors, including cement viscosity, multilevel OVCFs, and surgeon experience, were not analyzed. Further prospective, multicenter studies with large population and comprehensive predictors are required to verify and calibrate our findings.

## Conclusion

The incidence of overall BCLs in PKP for OVCFs combined with IVC was 37.6%, whereas intradiscal leakage was the most common type, developed in 32.1% of patients. CR and CVBF were identified as independent risk factors. A powered and well-calibrated predictive nomogram was established to accurately predict the probability of intradiscal leakage and further explore clinical strategies to prevent intradiscal leakage in such patients. Further prospective and multicenter studies are required to verify and calibrate our findings.

## Data Availability

The datasets generated during and/or analyzed during the current study are available from the corresponding author on reasonable request.
